# Report of a Successful Case of Trephining for Compression of the Brain

**Published:** 1852-01

**Authors:** J. W. H. Trugien

**Affiliations:** Portsmouth, Va.


*Stethoscope and Virginia Med. Gaz.*

### SURGERY.

*Report of a Successful Case of Trephining for Compression of the brain.* By J. W. H. Trugien, M.D., of Portsmouth, Va.—The report of the following case has been delayed until its result could be clearly established. It has ever been too much the custom with physicians to be hasty in the report of their cases—and many which have been published in the journals as successful, though in truth they were so, as far as their immediate result was concerned, have ultimately proved otherwise; and that, too, in some instances, it is to be feared, before the pages which contained the record of them had been given to the public. Such a course of proceeding is unfair, and detrimental to the true interests of our science, even though not so designed. The writer hopes, in the narration of his case, to avoid the same error. The subject of this report has so far recovered as to have resumed the active duties of life, being now engaged as a watchman at the railroad depot at this place. His general health is very good—even better, according to his own statement, than before the reception of the injury. It is but fair to state, however, that the wound of the operation is not yet entirely healed, a slight purulent discharge continuing, owing to exfoliation of the outer table of the skull. Several pieces have been removed at different times, and a small portion at the dressing of yesterday. Mental integrity has been completely restored, and the special senses (with the exception of that of sight) are unimpaired. Vision is not as good in the left eye as in the right. In the relation of the case I shall merely transcribe my notes made from day to day during its course.

June 25th, 1851.—Called to see J—— T——; occupation, laborer; aged 25; married. Stabbed in the head, a few minutes previously, with a knife; particular kind of knife not ascertained; situation of wound above left frontal eminence; sitting up; pale from loss of blood; no fracture or other lesion of bone discovered. Injury considered, and treated as a simple penetrating wound of the scalp.

26th.—Met patient on the street, feeling very comfortable.

27th.—Called to have wound dressed. Appearance good; union by first intention; feels comfortable; some restlessness, however, the preceding night; bowels unmoved since infliction of injury. ℞ Magnesiæ sulph. ℥j.

29th.—Again called to have wound dressed. Cicatrization complete, some uneasiness about the head, attributed (at the time) to excessive heat of weather and the thick growth of hair which covered the head, and which the patient refused to have shaved. It is proper to remark, in this place, that a medical friend present at the time thought he perceived some tumefaction below the wound and above the left eye. And as it will have some bearing upon the subsequent history of the case, mention must here be made of what was afterwards learned from the patient's wife, viz: that on his return home this day, he for the first time manifested some mental disturbance. On entering the house he remarked to her, that he should have to go out again to have his hair cut; and "I want, and I want," he slowly and hesitatingly continued to repeat, without the ability of telling what it was in reality he wanted. Getting worse and worse, he did not go out that day. From this date until July 2d, the history of the case is imperfect, as during this time he was under the treatment of another physician.

July 2d.—Saw patient again; found him in a half comatose condition; stupidity of expression; tumefaction below the wound; skin pitting on pressure; great disposition to sink back into the somnolent state after being aroused; pupils dilated; respiration slow, but unattended with stertor; great disposition to gape and yawn; pulse 60, full, slow, and laboring; bowels constipated, and urinary secretion deficient. The attending physician had very properly cupped him back of the neck, purged him, applied counter-irritants to the lower extremities, and placed him upon a mercurial course. No opening had been made into the swelling, from the impression that it was of an œdematous character. Hair had not been removed; recommended its removal.

July 7th.—Again a gap occurs in the history of the case until the above date, the patient not having been under the care of the writer during this time. Hair had been cut off, and for the first time another wound was discovered about an inch and a half above the first, and as nearly as possible over the coronal suture; surrounding tumefaction much reduced by the discharge of pus which was issuing from the wound. The introduction of the probe discovered the bone to be injured, the knife having penetrated it—to what distance, or whether it had passed through the diploe and internal table, could not then be ascertained.

*Symptoms.*—Great somnolence; stupid, vacant expression of countenance on being roused; much gaping and yawning; pupils dilated; some ptosis of left eye-lid; free discharge of pus; puffiness of scalp gone; no pain or uneasiness of head; respiration slow; no stertor; tongue protruded without difficulty—coated with a yellowish fur; pulse 60, full, slow, laboring; bowels constipated, and urinary secretion deficient; intellect much impaired; memory prominently implicated; patient unable to recall the names of even his wife and children; unable to tell whether he had eaten or drunk anything that day. A watch being showed him, he tells the hour, but not the minutes before or after a particular hour. Ordered one of the following pills every half hour, until free purging ensued:—℞. Ol. tiglii gttæ ij—micæ panis q. suf. Ft. Mass. Div. in pil. No. iv. Sinapisms to the back of the neck and lower extremities; egress of pus to be promoted by warm applications to the wound; light, unirritating diet.

8th. Symptoms same as yesterday; no action from Croton oil. Ordered following enema: ℞. Ol. tereb. ol. ricin. aa. ℥j.

9th. No improvement. Enema did not act, though thrice repeated. ℞. Hyd. chl. mit. gr.. xx., jalapæ pulv. gr. xxx., and repeat enemata.

10th. Much the same. Bowels twice moved.

11th. More rational to-day.

12th. Better to-day; understands questions, and replies with more readiness. Conversed with his wife, noticed his children, and recognized different individuals. Free discharge of pus from the wound. Pupils dilated, pulse 48, full and strong. Urine voluntarily discharged. Ate some light, nutritious food.

13th. Passed a bad night. Early in the evening restless and uneasy; tossing the head about, and then sinking back into a state of somnolency, amounting to almost complete insensibility—in which condition he was found at the visit of this morning. Utterly unconscious of what is going on around. Pupils dilated, and not responding to the action of the light; pulse full, slow, apoplectic, 44 in the minute. Inability to protrude the tongue. Wound discharging pus freely.

14th. No improvement. Operation of trephining determined upon, after a fair statement of its dangers to the family.

Four o'clock, P. M. Patient lies as if in *articulo mortis.* In the presence of Drs. Thos. Williamson, surgeon U. S. N., J. N. Schoolfield, Young, Maupin and Jas. Williamson of this place, Drs. Moore, Upshur and Wright of Norfolk city, and Dr. J. W. Garlick of King and Queen, the operation was performed, as follows: The hair having been shaved off to the extent of several inches around the wound, a crucial incision two inches in extent was made over it, and carried down to the bone. The hemorrhage from these incisions was inconsiderable. The crown of the trephine was then applied, and with it, for a guide, the pericranium was divided, by means of a scalpel, all around, so as to permit the teeth of the instrument to come in contact with the bone directly; the centre pin adjusted and the operation of boring commenced and gradually and carefully continued until the bone was completely drilled through and the button removed—whereupon a gush of purulent matter instantaneously issued from an opening in the dura mater, and continued to flow to the extent of an ounce and a half—the patient immediately emerging from his state of stupor and conversing with the by-standers. The pulse, which at the commencement of the operation was 44, at its close had mounted up to 80. Wound dressed with adhesive strips and compress, one end being left loose for the discharge of pus. Circular bandage also used to confine a compress over the middle temporal artery, which had been divided and was bleeding. My friend, Dr. J. Williamson, very kindly consented to remain with him during the night, which was passed quietly.

15th. Doing well; perfectly rational; pulse 70; has taken some light nourishment. Wound looking well; lightly dressed.

16th.—Quite comfortable; pulse 60; pus continues to flow from opening in dura mater. A probe introduced into this opening was passed down to the depth of two inches and a half by measurement, in an oblique direction, towards the anterior corner of the left lateral ventricle.

17th. Slept quietly last night; pulse 60; wound looking well.

18th. Removed ℥iss. of pus from abscess of the brain, by means of a small glass syringe, the nozzle of which was introduced into the opening through dura mater. Bowels confined. Ordered ol. ricini ℥ss.

19th. Removed half an ounce of pus from abscess.

20th. Doing well. Found patient sitting up smoking a pipe. From this date the patient continued to improve until the present time, when his condition is as described in the remarks prefatory to the case.

Remarks.—Injuries of the head have ever been subjects of greatest interest to the surgeon, as well from their frequent occurrence as from the obscure and anomalous symptoms occasioned by them. Owing to these latter circumstances, their diagnosis is complicated; prognosis rendered extremely fallible, and treatment undecided, and too often inefficient, if not actually hurtful. Nor is it so much to be wondered at, when we consider how obscure and unsettled are the phenomena of the brain and nervous system, even in their normal action. But when disturbed, as is most generally the case, by such injuries, they are rendered doubly obscure and unintelligible. Concussion and compression of the brain have ever engaged the most serious and earnest attention of the surgeon in their investigation; and although when well marked, there can be but little difficulty in diagnosing the one from the other, there are cases which puzzle the most discriminating. The two lesions may insensibly run into each other—what was concussion in the first instance becoming compression afterwards. Or there may be such a combination of symptoms that we are in doubt whether to call it a case of concussion or one of compression—the two morbid conditions seeming actually to co-exist. In the foregoing case the symptoms, though at first ill defined and obscure, were obviously due to compression of the brain. But what was the compressing force, was not equally clear. A portion of the internal table of the skull might have been detached by the force of the blow, and caused pressure upon the cranial mass. Or the same blow might have ruptured one or more of the delicate meningeal arteriolæ, and given rise to pressure from hemorrhage. Again—inflammation may have been lighted up in the meninges of the brain, and caused pressure, by the effusion of pus upon the cerebrum. In fact, some one or all of these reasons were adduced in explanation of the symptoms, by various medical gentlemen who saw the patient. However plausible, none of these explanations were satisfactory to the writer, who, early in the case, and before the second wound was discovered, referred them to penetration of the bony parietes of the skull, as well as the membranes, by which inflammation and subsequent suppuration had been occasioned. Hence, the gradual supervention of the symptoms. This opinion, which he is happy to state was concurred in by one or two of his confreres, received an ample confirmation by the result of the operation; for the button of bone removed by the trephine showed a cuneiform perforation of the third of an inch in length, as also the dura mater to the same extent. On reversing the button also, a spiculum of bone attached to its lower angle was discovered.

With regard to the treatment of this case, candor compels the admission that it might have been more actively antiphlogistic. But whether this would have at all conduced to a more favorable result, is very questionable. My own conviction is that it would not, and that the freest general depletion, the only part of the antiphlogistic course omitted, would have been unavailing in obviating the necessity of an operation. Still, I am free to admit that under the same circumstances, I would most copiously deplete from the general circulation, in order to give the patient every chance of recovering, without exposing him to the risk of such an operation, which though successful in instances innumerable, is always attended with much danger to the patient, and, in a multitude of cases, fatal, either from the supervention of acute inflammation of the brain and its meninges, or the more remote consequences of hernia cerebri. No case in medicine or surgery, however simple, can fail, if closely studied, to afford useful and instructive practical lessons. The study of this case has been replete with such to the writer. It adds another to the many recorded instances of severe injury to the brain, from which recovery has taken place under seemingly the most unfavorable circumstances, and encourages us never to despair of affording relief even at the last extremity. A volume might be filled with cases illustrative of what has been said. The recorded experience of army surgeons upon this point is indeed almost incredible, but for the undoubted veracity of the writers. The work of Mr. Guthrie on "Injuries of the Head," is full of cases of this kind. The conclusions of this surgeon, after the enumeration of many cases in support thereof, are that "injuries of apparently equal extent are more dangerous on the *forehead* than on the side or middle of the head, and much less so on the back part than on the side." He further remarks: "I have never seen a person live with a foreign body lodged in the *anterior* lobe of the brain, although I have seen several recover with a loss of a portion of the brain at this point." The medical journals of the day have contained the records of recovery from the most extensive and terrible injuries of the head. Dr. Harlow's case of "recovery from the passage of an iron bar through the head," (*vide* American Journal of Medical Science, July, 1850,) must be familiar to every professional reader. Physiological experiments too, as those of Flourens, Majendie, Mayo and others, clearly demonstrate that the most severe injuries may be inflicted upon the brain with comparative impunity, and that it may be even sliced away to a very considerable extent, without destroying the animal.

Without protracting these remarks, I would, in conclusion, call attention to two practical points connected with the case. Mention has been made in the detail of the case of the use of the syringe in evacuating the abscess of the brain of the pus. Mere position would not have accomplished this, as it was fairly tried. Suction, by the instrument, however, was entirely successful in the removal of every particle of pus. The writer remembers having heard this plan recommended by Dr. William Gibson, of the University of Pennsylvania, and can bear testimony to its efficiency.—*Stethoscope and Virginia Med. Gaz.*

